# Overexpression of *DgWRKY4* Enhances Salt Tolerance in Chrysanthemum Seedlings

**DOI:** 10.3389/fpls.2017.01592

**Published:** 2017-09-13

**Authors:** Ke Wang, Yin-Huan Wu, Xiao-Qin Tian, Zhen-Yu Bai, Qian-Yu Liang, Qing-Lin Liu, Yuan-Zhi Pan, Lei Zhang, Bei-Bei Jiang

**Affiliations:** Department of Ornamental Horticulture, Sichuan Agricultural University Chengdu, China

**Keywords:** WRKY transcription factor, *DgWRKY4*, salt stress, transgenic chrysanthemum, gene expression

## Abstract

High salinity seriously affects the production of chrysanthemum, so improving the salt tolerance of chrysanthemum becomes the focus and purpose of our research. The WRKY transcription factor (TF) family is highly associated with a number of processes of abiotic stress responses. We isolated *DgWRKY4* from *Dendranthema grandiflorum*, and a protein encoded by this new gene contains two highly conserved WRKY domains and two C_2_H_2_ zinc-finger motifs. Then, we functionally characterized that *DgWRKY4* was induced by salt, and *DgWRKY4* overexpression in chrysanthemum resulted in increased tolerance to high salt stress compared to wild-type (WT). Under salt stress, the transgenic chrysanthemum accumulated less malondialdehyde, hydrogen peroxide (H_2_O_2_), and superoxide anion ($O_{2}^{-}$) than WT, accompanied by more proline, soluble sugar, and activities of antioxidant enzymes than WT; in addition, a stronger photosynthetic capacity and a series of up-regulated stress-related genes were also found in transgenic chrysanthemum. All results demonstrated that *DgWRKY4* is a positive regulatory gene responding to salt stress, via advancing photosynthetic capacity, promoting the operation of reactive oxygen species-scavenging system, maintaining membrane stability, enhancing the osmotic adjustment, and up-regulating transcript levels of stress-related genes. So, *DgWRKY4* can serve as a new candidate gene for salt-tolerant plant breeding.

## Introduction

High salinity significantly limits the growth and productivity of plants worldwide. To adapt to high salinity environment, plants have developed a set of elaborate and intricate mechanisms. At the molecular level, the induced transcription factors (TFs) such as AP2/EREBP, WRKY, MYB, and bHLH play an important role in activating downstream stress-responsive genes to protect plants from salt stress persecution (Chinnusamy et al., 2006; Hennig, 2012). The WRKY TF is a big and valuable family of regulatory proteins in plants (Rushton et al., 2012). Since the first WRKY TF was identified in sweet potato (Ishiguro and Nakamura, 1994), some of the other WRKY genes were also been successively characterized in other species. All the WRKY proteins contain one or two DNA-binding domains consisting of 60 amino acid regions with the highly conserved sequence WRKYGQK at its N-terminus and a zinc-finger motif (C-X_4-5_-C-X_22-23_-H-X-H or C-X_7_-C-X_23_-H-X-C) at C-terminus (Eulgem et al., 2000; Rushton et al., 2010). WRKYGQK motif may be replaced by WRKYGKK, WRKYGEK, WRKYGSK, or WRKYDQK in some plant species (Xiu et al., 2016). The WRKY proteins can fall into three groups, group I proteins contain two WRKY domains with C-X_4-5_-C-X_22-23_-H-X-H zinc-finger motifs, group II proteins just contain one WRKY domain with a C-X_4-5_-C-X_22-23_-H-X-H motif, and group III proteins contain one WRKY domain with a C-X_7_-C-X_23_-H-X-C motif.

WRKY TFs can positively or negatively regulate downstream-related genes and play roles in multiple processes of plants, such as seed development (Johnson et al., 2002), leaf senescence (Miao et al., 2004), and confrontation with stresses (Xie et al., 2005; Ryu et al., 2006; Eulgem and Somssich, 2007; Pandey and Somssich, 2009; Sun et al., 2013). According to previous reports, genes encoding WRKY TFs can be induced by NaCl, cold, drought, salicylic acid (SA), ethylene (ET), abscisic acid (ABA), methyl jasmonate (MeJA), and hydrogen peroxide (H_2_O_2_) (Wang et al., 2013; Zhou et al., 2015; Xiu et al., 2016). So far, overexpression of some WRKY genes has successfully enhanced plants tolerance to several abiotic stresses. For example, overexpressing *OsWRKY11* improved high temperature and salt tolerance of overexpressed lines (Wu et al., 2009). Overexpressing cotton genes *GhWRKY17*, *GhWRKY34*, and *GhWRKY41* increased salt and drought tolerance of transgenic *Nicotiana benthamiana* (Yan et al., 2014; Chu et al., 2015; Zhou et al., 2015). Moreover, overexpressing wheat genes *TaWRKY19* and *TaWRKY93* in *Arabidopsis* enhanced its tolerance to salt and drought (Niu et al., 2012; Qin et al., 2015). These genes conferred plants tolerance to abiotic stresses primarily through scavenging reactive oxygen species (ROS), improving the osmotic adjustment, maintaining membrane stability, maintaining the Na^+^/K^+^ homeostasis, regulating ABA signaling, and activating the stress-related genes.

Chrysanthemum is a kind of cut flower with great ornamental value. However, its production is severely affected by high salinity. *CmWRKY1* and *CmWRKY10* were reported to enhance the drought tolerance of chrysanthemum through an ABA-mediated pathway (Fan et al., 2016; Jaffar et al., 2016). In contrast, *CmWRKY17* negatively regulates salt tolerance in transgenic chrysanthemum (Li et al., 2015). We have previously isolated three WRKY genes (*DgWRKY1*, *DgWRKY3*, and *DgWRKY5*) and characterized that they could confer salt tolerance to tobacco or chrysanthemum (Liu et al., 2013, 2014; Liang et al., 2017). But the study on WRKY family of chrysanthemum is still incomplete. In order to analyze WRKY family of chrysanthemum in multiple angles and complement its information, as well as provide more selections of excellent genes for improving salt tolerance of chrysanthemum, we isolated and functionally characterized *DgWRKY4* gene. Overexpressing *DgWRKY4* in chrysanthemum resulted in increased tolerance to high salt stress compared to wild-type (WT), indicating that *DgWRKY4* can serve as a new candidate gene for salt-tolerant plant breeding.

## Materials and Methods

### Plant Materials and Treatments for Analyses of *DgWRKY4* Expression Pattern

Seedlings of WT Chrysanthemum cv. Jinba were cultured in the incubator, setting the condition as 25°C/16 h light and 22°C/8 h dark cycles, light intensity of 200 μmol m^-2^ s^-1^, and relative humidity of 70%. Seedlings with six to seven leaves were treated with 200 mM NaCl solutions, and leaves were harvested at several times after treatment, frozen in liquid nitrogen immediately, and stored at -80°C. Roots, stems, and leaves of the same untreated seedlings were collected for tissue-specific expression analyses.

### Analysis of Gene Expression Levels

*DgWRKY4* expression level was monitored by quantitative real-time polymerase chain reaction (qRT-PCR) using the SsoFast EvaGreen supermix (Bio-Rad, Hercules, CA, United States) and Bio-Rad CFX96^TM^ detection system. *EF1*α as the internal reference, the 20 μL qRT-PCR reaction mixture was incubated under the following program: 30 s at 95°C for 1 cycle, then 15 s at 95°C and 30 s at 60°C for 40 cycles, and a single melt cycle from 65 to 95°C in the end. Each reaction was set with three repetitions. Final relative expression levels were calculated by the 2^-ΔΔ*C*_T_^ method. The primers used in qRT-PCR are listed in **Table 1**.

**Table 1:** Primers used in this study.

	Forward primers	Reverse primers
**Primers used for cloning of *DgWRKY4***		
*DgWRKY4*	TAAATATAACTTTCTCAAACACATCCT	GACCCTACATATATGTACATCAACAC
**Primers used to qRT-PCR**		
*DgWRKY4*	CTCAAACACATCCTACAAATTCCC	AGAAATGGGAAGTGAAGGTGG
*EF1a*	TTTTGGTATCTGGTCCTGGAG	CCATTCAAGCGACAGACTCA
*DgCuZnSOD*	CCATTGTTGACAAGCAGATTCCACTCA	ATCATCAGGATCAGCATGGACGACTAC
*DgCAT*	TACAAGCAACGCCCTTCAA	GACCTCTGTTCCCAACAGTCA
*DgAPX*	GTTGGCTGGTGTTGTTGCT	GATGGTCGTTTCCCTTAGTTG
*DgP5CS*	TTGGAGCAGAGGTTGGAAT	GCAGGTCTTTGTGGGTGTAG
*DgDREB1A*	CGGTTTTGGCTATGAGGGGT	TTCTTCTGCCAGCGTCACAT
*DgDREB2A*	GATCGTGGCTGAGAGACTCG	TACCCCACGTTCTTTGCCTC
*DgCSD1*	TTCGTCCATCAGTCTAGTATCAAG	ATCACCACCACCACCACCTC
*DgCSD2*	AGTGAAGATGGACGAAAAAAGG	CTAGCAAAATGACCAACCCG


### Salt Treatment of Transgenic Chrysanthemum and Stress Tolerance Assays

For salt treatment, two overexpressed lines (OE-4 and OE-6) and WT of chrysanthemum were planted to a mixture of peat and perlite, then cultured in a light incubator (25°C/16 h light and 22°C/8 h dark cycles). Soil-grown chrysanthemum seedlings at six to seven leaves stage were irrigated with an increased concentration of NaCl solution: 100 mM for 1–5 days (d), 200 mM for 6–10 days, and 400 mM for 11–15 days, using Chen et al. (2012) as a reference. Under salinity conditions, leaves four to five from buts were harvested at 0, 5, 10, and 15 days for physiological and molecular experiments in subsequent. Survival rates were calculated after 2 weeks of recovery.

### Determination of Physiological Indexes and Leaf Gas Exchange Parameters

Leaves of seedlings were used for measurements. Activities of superoxide dismutase (SOD), peroxidase (POD), and catalase (CAT) were measured following Beauchamp and Fridovich (1971),Ranieri et al. (2000), and Zhang L. et al. (2011), respectively. Malondialdehyde (MDA) content in chrysanthemum was measured according to Zhang et al. (2009). Accumulation of proline was measured following Irigoyen et al. (1992) and soluble sugar following Wang et al. (2013). The chlorophyll content was detected following Huang et al. (2010). Leaf gas exchange parameters were measured following Mguis et al. (2013), setting the endogenous light intensity was 600 μmol m^-2^ S^-1^, the concentration of CO_2_ was 360 μL L^-1^, and the temperature was 25°C.

### Histochemical Detection of Reactive Oxygen Species (ROS)

Leaves of chrysanthemum plants were performed with histochemical staining to detect the accumulation of H_2_O_2_ and superoxide anion ($O_{2}^{-}$) using 3,3′-diaminobenzidine (DAB) and nitroblue tetrazolium (NBT), respectively. Detached leaves were soaked in 1 mg mL^-1^ DAB or NBT solution under illumination. When brown or blue spots appeared, leaves were bleached by 95% ethanol. Finally, photos were taken. In addition, the H_2_O_2_ and $O_{2}^{-}$ concentration were determined by detection kits (Nanjing Jiancheng Bioengineering Institute, China).

### Expression of Stress-Response Genes in *DgWRKY4* Transgenic Chrysanthemum

The RNA of both transgenic chrysanthemum and WT was extracted and reversed to cDNA as described above. Then expressions of stress-response genes in transgenic chrysanthemum were detected by qRT-PCR. *DgCuZnSOD*, *DgCAT*, *DgAPX*, *DgP5CS*, *DgDREB1A*, *DgDREB2A*, *DgCSD1*, and *DgCSD2* were monitored, using *EF1*α as the internal reference. All relevant primers of qRT-PCR are listed in **Table 1**.

### Statistical Analysis

All experiments were performed for three biological repeats, and means and standard errors were calculated for the variables comparison. All data were analyzed by SPSS version 20.0 (IBM Corporation) at a significant level of 0.05.

## Results

### *DgWRKY4* Cloning and Generation of Transgenic Chrysanthemum

Using high-throughout sequencing technique, we obtained the transcriptome database of chrysanthemum under salinity condition. From the database, a large number of salt-induced transcripts were identified, and *DgWRKY4* is one of them with significantly induced by salinity. Total RNA extraction of chrysanthemum leaves was performed by TRIzol Reagent (Mylab, Beijing, China). The full-length cDNA of *DgWRKY4* was obtained by PCR, then inserted into pCAMBIA 2300 with the control of cauliflower mosaic virus (CaMV) 35S promoter. The vector was transformed into chrysanthemum by *Agrobacterium tumefaciens* (strain LBA4404) (An et al., 1988). *DgWRKY4* high expression lines OE-4 and OE-6 were selected for subsequent experiments.

### Sequence Analysis of DgWRKY4

**FIGURE 1:**
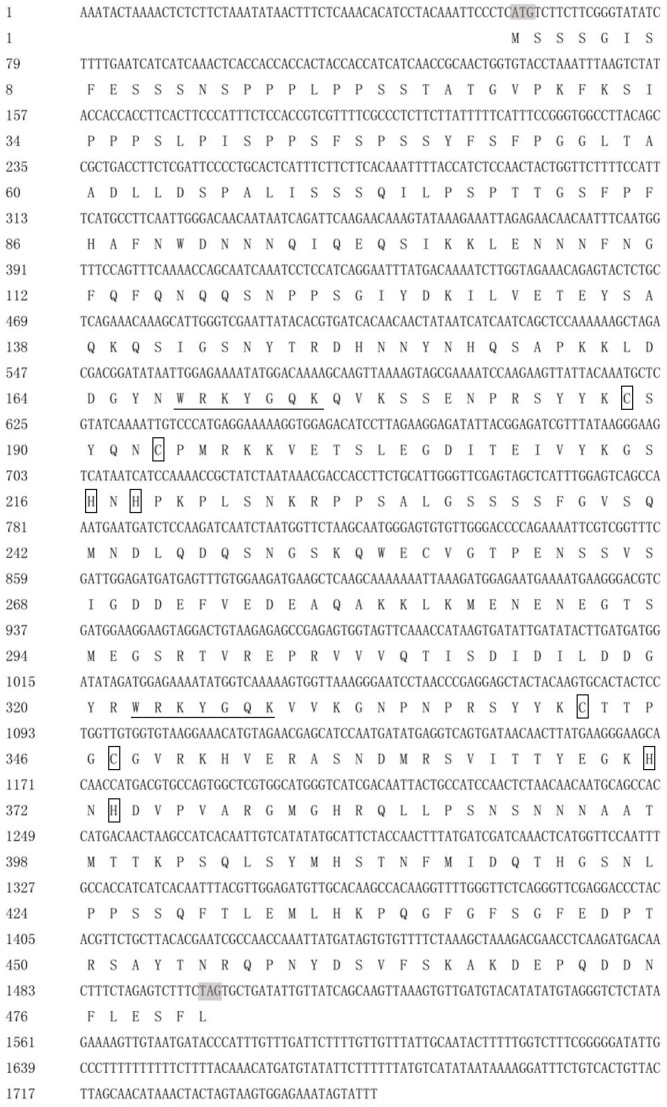
Nucleotide and deduced amino acid sequences of *DgWRKY4*. The WRKY domain is underlined. The two cysteines and two histidines in the zinc-finger motifs are boxed.

**FIGURE 2:**
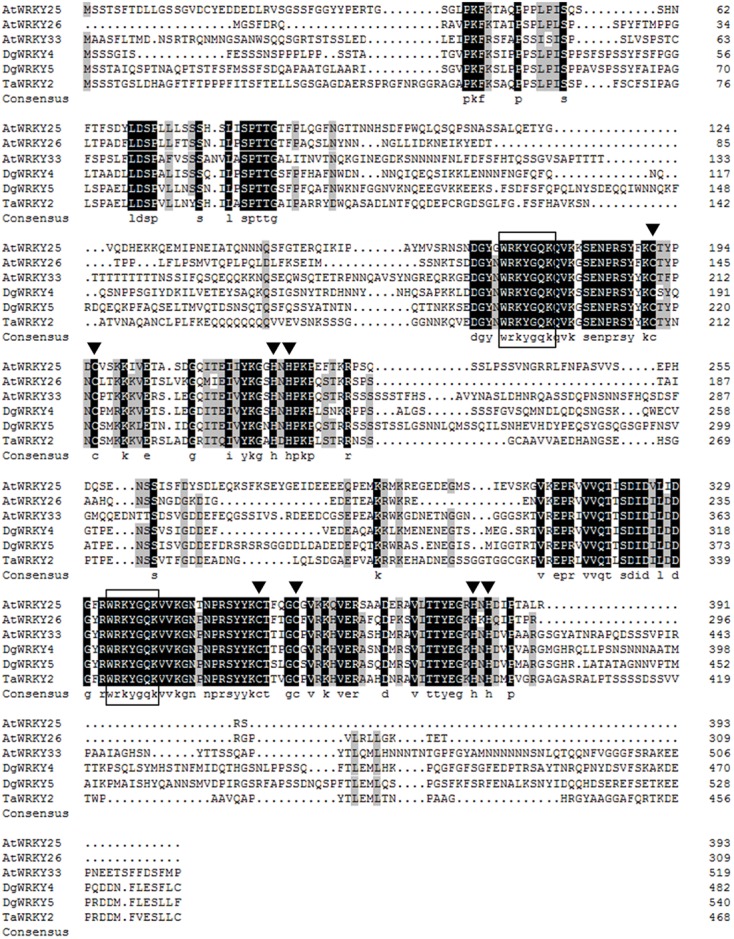
Sequence alignment of the deduced DgWRKY4 protein with known homologs. The comparison was conducted by DNAMAN (version 6.0). Amino acid residues conserved in all five sequences are shaded in black, and those conserved in four sequences are shaded in light gray. The completely conserved WRKYGQK amino acids are boxed. The cysteines and histidines in zinc-finger motifs are indicated by arrowheads (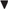). *Arabidopsis thaliana* (AtWRKY25, NP_180584; AtWRKY26, AAK28309; AtWRKY33, NP_181381) and *Triticum aestivum* (TaWRKY2, EU665425).

**FIGURE 3:**
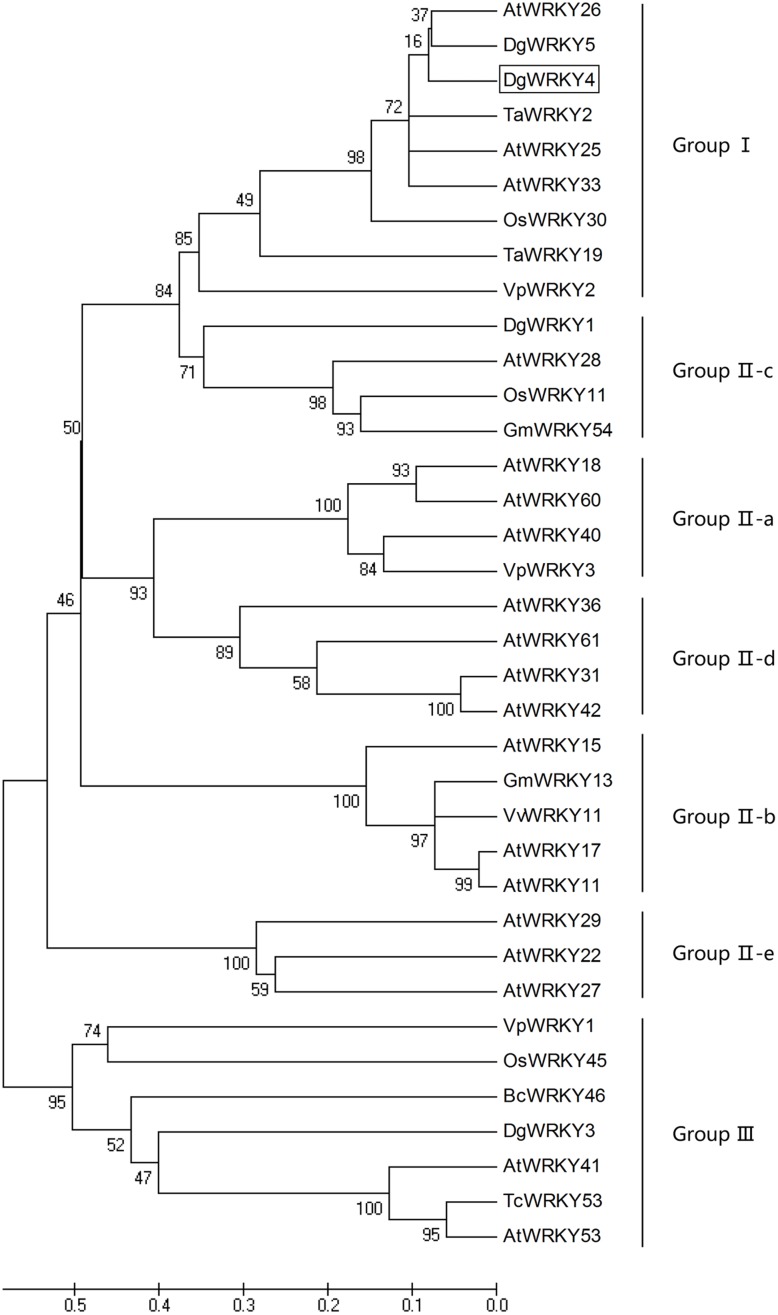
Phylogenetic tree analysis of DgWRKY4 and WRKY proteins from different species. The amino acid sequences of the conserved WRKY domain region were subjected to the Bootstrap test of phylogeny by the MEGA program (ver. 5). DgWRKY4 is boxed. The plant WRKY proteins used for the phylogenetic tree are as follows: DgWRKY1 (KC153303), DgWRKY3 (KC292215), DgWRKY5 from *Dendranthema grandiflorum*; VpWRKY1 (GQ884198), VpWRKY2 (GU565706), VpWRKY3 (JF500755) from *Vitis pseudoreticulata*; VvWRKY11 (EC935078) from *Vitis vinifera*; BcWRKY46 (HM585284) from *Brassica campestris*; TcWRKY53 (EF053036) from *Thlaspi caerulescens*; TaWRKY2 (EU665425), TaWRKY19 (EU665430) from *Triticicum aestivum*; GmWRKY13 (DQ322694), GmWRKY54 (DQ322698) from *Glycine max*; OsWRKY11 (AK108745), OsWRKY30 (NP_001062148), OsWRKY45 (AY870611) from *Oryza sativa*; AtWRKY11 (NP_849559), AtWRKY15 (NP_179913.1), AtWRKY17 (NP_565574.1), AtWRKY18 (NP_567882), AtWRKY22 (AEE81999), AtWRKY25 (NP_180584), AtWRKY26 (AAK28309), AtWRKY27 (NP_568777), AtWRKY28 (NP_193551), AtWRKY29 (AEE84774), AtWRKY31 (NP_567644), AtWRKY33 (NP_181381), AtWRKY36 (NP_564976), AtWRKY40 (NP_178199), AtWRKY41 (NP_192845), AtWRKY42 (NP_192354), AtWRKY53 (NP_194112), AtWRKY60 (NP_180072), AtWRKY61 (NP_173320) from *Arabidopsis thaliana*.

DgWRKY4 contained a complete open-reading frame (ORF) of 1534 bp encoding a putative protein of 482 amino acids with a predicted protein molecular weight of 53.6 kDa (**Figure 1**). Multiple alignment between DgWRKY4 and other four WRKY proteins by DNAMAN showed that DgWRKY4 contained two WRKY domains of WRKYGQK and two C_2_H_2_ zinc-finger motifs (C-X_4_-C-X_22_-H-X-H and C-X_4_-C-X_23_-H-X-H) (**Figure 2**). Based on the classification method (Rushton et al., 2010; **Figure 3**), phylogenetic analysis showed that DgWRKY4 was clustered into group I of the WRKY family and most closely related to AtWRKY25, AtWRKY26, AtWRKY33, DgWRKY5, and TaWRKY2.

### Expression of *DgWRKY4* Is Regulated by Salt Stress

**FIGURE 4:**
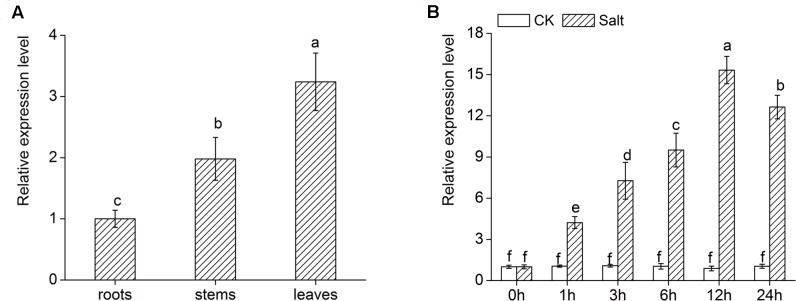
Expression of *DgWRKY4* in different organs of WT chrysanthemum and under salt stress. **(A)** Expression patterns of *DgWRKY4* in roots, stems, and leaves of WT chrysanthemum under normal condition. **(B)** *DgWRKY4* expression of WT chrysanthemum leaves in response to 200 mM NaCl treatment. CK means non-stress conditions. Data represent means and standard errors of three replicates. The different letters above the columns indicate significant differences (*P* < 0.05) according to Duncan’s multiple range test.

*DgWRKY4* expression of different tissues was measured by qRT-PCR to figure out its expression pattern in chrysanthemum. As shown in **Figure 4A**, there was higher transcript abundance of *DgWRKY4* in leaves than in stems and roots. In addition, the expression of *DgWRKY4* in WT chrysanthemum leaves was gradually increased up to 12 h after treatment with 200 mM NaCl (**Figure 4B**). This demonstrated that the *DgWRKY4* was induced by salinity.

### *DgWRKY4* Overexpression Enhances Chrysanthemum Salt Tolerance

**FIGURE 5:**
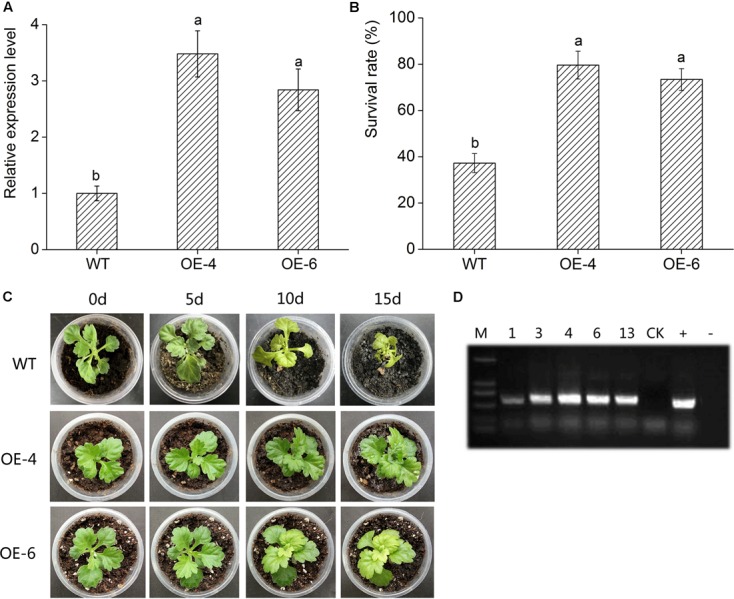
Overexpression of *DgWRKY4* in transgenic chrysanthemum resulted in enhanced tolerance to salt stress. **(A)** Transcript levels of *DgWRKY4* in WT and transgenic chrysanthemum. *EF1*α serves as the internal reference and error bars based on three replicates. **(B)** The survival rates of overexpressed lines and WT after 2 weeks recovery. **(C)** Phenotypic comparison of *DgWRKY4* overexpressed lines (OE-4 and OE-6) and WT under salt stress. **(D)** PCR analysis of *DgWRKY4* transgenic chrysanthemum lines. Data represent means and standard errors of three replicates. The different letters above the columns indicate significant differences (*P* < 0.05) according to Duncan’s multiple range test.

*DgWRKY4* transcript levels of two transgenic lines were measured through qRT-PCR. The result showed that the *DgWRKY4* transcript level of lines OE-4 and OE-6 was distinctly (*P* < 0.05) higher than that of WT (**Figure 5A**), therefore these two lines were selected for further salt-tolerance researches. Under normal conditions, all chrysanthemum showed no obvious phenotypic difference at the seedling stage (data not shown). Under salt stress, leaves of WT plants were yellowed and wilted, while transgenic chrysanthemum’s remained green (**Figure 5C**). Moreover, after 2 weeks of recovery from salt stress, the survival percentage of OE-4 and OE-6 was 73.4% and 79.6%, respectively, whereas WT plants’ was 35.23% (**Figure 5B**).

### Analyses of Chlorophyll Content and Photosynthesis Under Salt Stress

**FIGURE 6:**
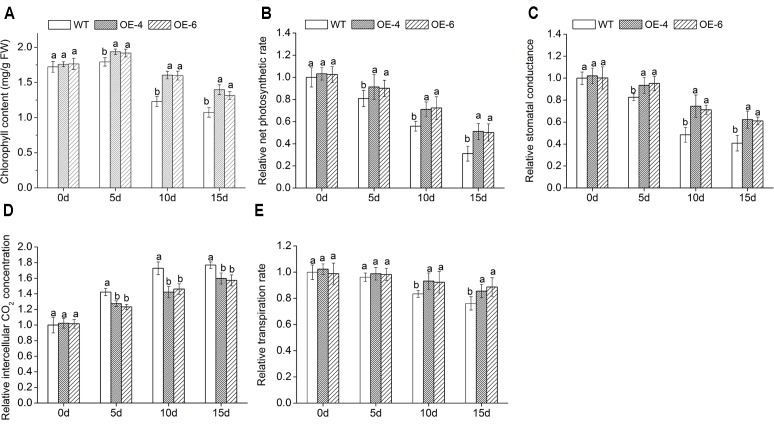
Assay of leaf gas exchange parameters in overexpressed lines and WT under salt stress. **(A)** Chlorophyll content. **(B)** Pn. **(C)** Gs. **(D)** Ci. **(E)** Tr. Data represent means and standard errors of three replicates. The different letters above the columns indicate significant differences (*P* < 0.05) according to Duncan’s multiple range test.

When exposed to salt conditions, the chlorophyll content of overexpressed lines was remarkably (*P* < 0.05) higher than WT (**Figure 6A**), suggesting that transgenic chrysanthemum was better able to maintain their chlorophyll than WT. In addition, we measured leaf gas exchange parameters. With the increase of NaCl concentration, the net photosynthetic rate (Pn), stomatal conductance (Gs), and transpiration rate (Tr) decreased in all lines, while intercellular CO_2_ concentration (Ci) increased, but reduction and increase degree of overexpressed lines were clearly (*P* < 0.05) smaller than WT (**Figures 6B–E**). It suggested that photosynthesis of transgenic chrysanthemum was less inhibited by salt stress than WT.

### Overexpression of *DgWRKY4* Reduces ROS Accumulation and Oxidative Damage

**FIGURE 7:**
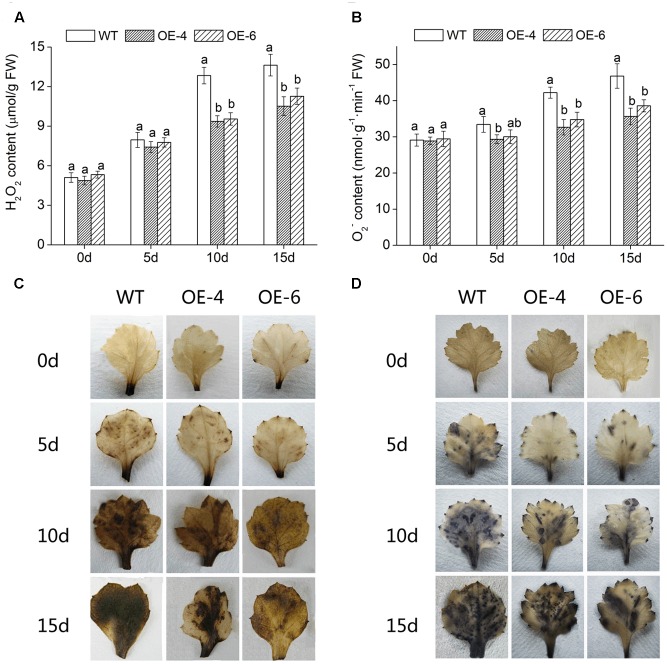
Analysis of ROS accumulation levels in WT and *DgWRKY4* overexpressed chrysanthemum lines (OE-4 and OE-6) under salt stress. **(A,B)** Quantitative measurement of H_2_O_2_ and $O_{2}^{-}$ in WT, OE-4, and OE-6 after 0, 5, 10, and 15 days of exposure to salinity. **(C,D)** Histochemical staining with DAB and NBT for assessing the accumulation of H_2_O_2_ and $O_{2}^{-}$, respectively, under non-stress and salt conditions. Data represent means and standard errors of three replicates. The different letters above the columns indicate significant differences (*P* < 0.05) according to Duncan’s multiple range test.

**FIGURE 8:**
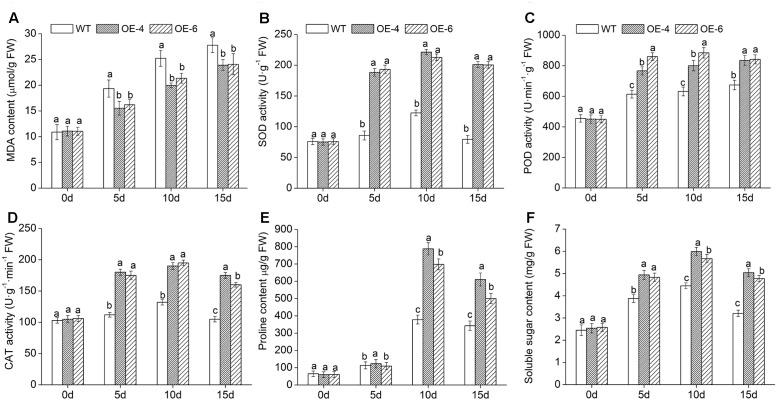
Physiological effects of salt stress on WT and *DgWRKY4* overexpressed chrysanthemum lines. **(A)** Leaf MDA content. **(B)** Leaf SOD activity. **(C)** Leaf POD activity. **(D)** Leaf CAT activity. **(E)** Leaf proline content. **(F)** Leaf soluble sugar content. Data represent means and standard errors of three replicates. The different letters above the columns indicate significant (*P* < 0.05) differences according to Duncan’s multiple range test.

To intuitively understand the oxidation status of chrysanthemum, the accumulation of two major ROS (H_2_O_2_ and $O_{2}^{-}$) was detected with DAB staining and NBT staining. Histochemically, staining showed that WT accumulated more H_2_O_2_ and $O_{2}^{-}$ than two overexpressed lines (OE-4 and OE-6), as less brown or blue spots were observed in overexpressed lines (**Figures 7C,D**). In addition, quantitative analysis also showed that H_2_O_2_ and $O_{2}^{-}$ levels in leaves of all lines were increased after exposure to salt condition, whereas WT significantly (*P* < 0.05) accumulated more H_2_O_2_ and $O_{2}^{-}$ than transgenic chrysanthemum (**Figures 7A,B**). Similarly, under salt stress, the MDA (the end product of lipid oxidation) accumulation level was significantly (*P* < 0.05) lower in overexpressed lines than in WT (**Figure 8A**). As a result, the accumulation of ROS in *DgWRKY4*-overexpression chrysanthemum was less than WT, indicating that *DgWRKY4* reduced the ROS levels and alleviated the oxidative damage under salinity condition.

### Physiological Changes in *DgWRKY4* Transgenic Chrysanthemum

To investigate the underlying cause of the decreased ROS (H_2_O_2_ and $O_{2}^{-}$) accumulation in transgenic chrysanthemum under salinity condition, activities of three symbolic antioxidant enzymes (SOD, POD, and CAT) were monitored at various time points. Under normal condition, no significant difference of these three enzymes activities was observed between WT and overexpressed lines. Upon exposure to salt stress, there was a certain degree of increases in all lines; furthermore, these increases were remarkably (*P* < 0.05) greater in overexpressed lines than in WT (**Figures 8B–D**). The above showed that overexpressing *DgWRKY4* conferred transgenic chrysanthemum higher antioxidant enzyme activities to against ROS persecution.

Subsequently, we monitored changes of proline and soluble sugar content to explore the regulation of osmotic mechanism in *DgWRKY4* transgenic chrysanthemum under salt stress. Compared with WT, overexpressed lines accumulated remarkably (*P* < 0.05) higher levels of proline and soluble sugar (**Figures 8E,F**) under salinity condition. These data suggested that overexpression of *DgWRKY4* conferred transgenic chrysanthemum higher osmotic pressure to cope with the dehydration stress evoked by salt stress.

### The Molecular Mechanism of *DgWRKY4* Overexpression Promoting Salt Tolerance

**FIGURE 9:**
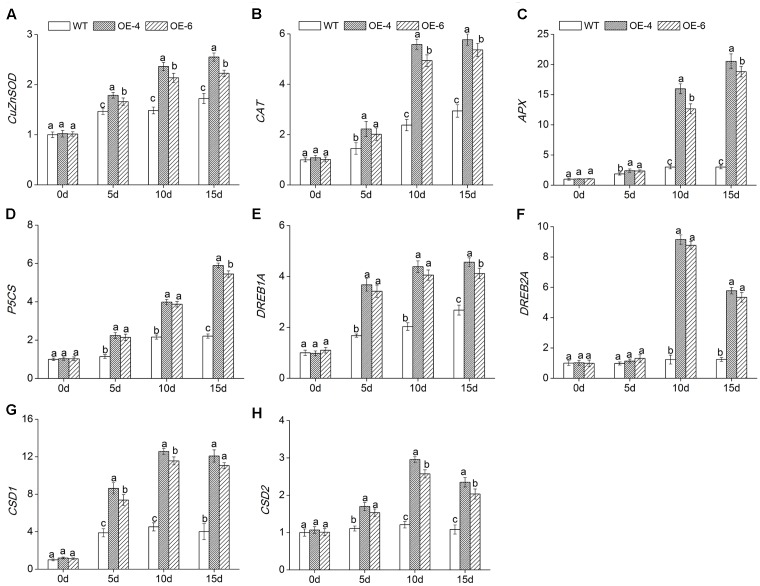
Expression of stress-related genes in WT and overexpressed lines (OE4 and OE-6) at various time points (0, 5, 10, and 15 days) of salinity. **(A)** *DgCu/ZnSOD*. **(B)** *DgCAT*. **(C)** *DgAPX*. **(D)** *DgP5CS*. **(E)** *DgDREB1A*. **(F)** *DgDREB2A*. **(G)** *DgCSD1*. **(H)** *DgCSD2. EF1a* was amplified as a control. Data represent means and standard errors of three replicates. The different letters above the columns indicate significant (*P* < 0.05) differences according to Duncan’s multiple range test.

To reveal the molecular mechanism of enhanced salt tolerance in *DgWRKY4*-overexpression chrysanthemum, expressions of eight abiotic stress-response genes were detected by qRT-PCR. Under normal condition, these eight gene expression levels were not different in all lines. Under salt treatment, the transcript accumulation of *DgCuZnSOD*, *DgCAT*, and *DgAPX*, which encode ROS-scavenging enzymes, and *DgP5CS*, which functions in osmotic adjustment, was increased remarkably (*P* < 0.05) in overexpressed lines compared to WT. The transcription levels of above four genes in overexpressed chrysanthemum reached a maximum by day 15, as they were about 1.39-, 1.89-, 6.54-, and 2.57-fold greater than in WT (**Figures 9A–D**). Moreover, the other four genes, such as *DgDREB1A*, *DgDREB2A*, *DgCSD1*, and *DgCSD2*, were all significantly (*P* < 0.05) up-regulated in overexpressed lines than WT under salinity condition. Especially by day 10, the transcription levels of above four genes in overexpressed lines were averagely 2.08-, 7.27-, 2.67-, and 2.28-fold greater than in WT (**Figures 9E–H**). Our data suggested that *DgWRKY4* overexpression may promote salt tolerance via up-regulating expression levels of genes which involved in controlling signaling pathways and function in scavenging excess ROS and relieving osmotic stress.

## Discussion

Chrysanthemum is an ornamental flower widely used in China, but its production is severely affected by salt stress. For improving the salt tolerance of chrysanthemum, we over expressed a salt-induced gene *DgWRKY4* in chrysanthemum. And the final experimental results proved that overexpression of *DgWRKY4* could enhance salt tolerance of chrysanthemum without growth abnormality. Currently, our study on chrysanthemum seedlings is temporarily limited to the laboratory, and whether the production of transgenic chrysanthemum can be improved under salt stress needs to be further explored and verified in field experiments.

The members in the same group may have similar functions. Previous studies have been reported that *AtWRKY25* and *AtWRKY33* overexpression could increase plants salt tolerance (Jiang and Deyholos, 2009); overexpression of *TaWRKY2* conferred transgenic *Arabidopsis* with higher salt and drought tolerance (Niu et al., 2012); and overexpression of *DgWRKY5* enhanced salt tolerance in transgenic chrysanthemum (Liang et al., 2017). Since DgWRKY4, AtWRKY25, AtWRKY33, TaWRKY2, and DgWRKY5 all belong to the group I of the WRKY family, we inferred that DgWRKY4 may have a positive effect on salt stress. Moreover, our previous researches proved that DgWRKY1 and DgWRKY3, which, respectively, belong to group II-c and group III, were also two positive regulator of salt tolerance (Liu et al., 2013, 2014). It can be speculated that the WRKY family plays an important role in salt stress resistance.

Transcription factors usually act as “master switches,” since they mainly enhance plants stress tolerance by activating and regulating the expression of downstream genes to adapt to the coercive environment (Liu et al., 1998). DREB1 and DREB2 belong to AP2/EREBP TF family (Shinozaki and Yamaguchi-Shinozaki, 2000), and overexpressing drought response element binding (DREB) protein genes has been reported to positively regulate downstream stress-responsive genes and confer increased tolerance of drought, high salinity, or low temperature to transgenic plants (Yamaguchi-Shinozaki and Shinozaki, 2001; Oh et al., 2005; Cong et al., 2008; Zhang et al., 2013; Chen et al., 2016). Cold shock domain proteins (CSDs) ordinarily were regarded as working on conferring cold tolerance to plants (Chaikam and Karlson, 2008; Park et al., 2009), however, Kim et al. (2013) reported that overexpression of *AtCSP3* (encoding one member of CSD TF family) could enhance tolerance to salt and drought stresses in *Arabidopsis*. In our study, *DgDREB1A*, *DgDREB2A*, *DgCSD1*, and *DgCSD2* were all up regulated greater in overexpressed lines than in WT (**Figures 9E–H**), indicating that overexpression of *DgWRKY4* could actively promote the expression of these stress-inducible TFs, then further activate more downstream genes participating in many vital biological processes. In addition, the DREB family is mainly responding to drought stress. The up regulation of *DgDREB1A/2A* caused by overexpression of *DgWRKY4* let us infer that transgenic chrysanthemum may be conferred the drought tolerance. And additional work is also needed to understand the molecular mechanism of *DgWRKY4* in drought stress response.

Photosynthesis is the most important factor in plant productivity, and chlorophyll is an essential factor in the process of photosynthesis. Under salinity condition, chlorophyll content of WT reduced more rapidly than transgenic chrysanthemum, which was consistent with the phenomenon that WT turning yellowed and wilted was faster than transgenic chrysanthemum. Decrease of chlorophyll content mainly due to salt stress increased the chlorophyll enzymes activity and promoted chlorophyll degradation (Yeo, 1998). Salt stress also could cause leaf water potential and stomatal conductance decrease, limiting CO_2_ to photosynthetic mechanism, thus inhibiting photosynthesis (Mguis et al., 2013). However, in our study, the decrease of Pn, Gs, and Tr, and the increase of Ci suggested that non-stomatal restriction was a major factor in the Pn decline of chrysanthemum under high salinity conditions (100–400 mM). Possible reasons for this include an increase of the resistance of mesophyll cells to stomata diffusion, a decrease of CO_2_ solubility, a decreased affinity of Rubisco enzyme to CO_2_, a decreased RuBP regenerative capacity, or the stability of key components in photosynthetic apparatus was decreased by salt stress (Zheng et al., 2002). Leaf gas exchange parameters attested that transgenic chrysanthemum had stronger photosynthesis than WT under salt stress, indicating *DgWRKY4* may play a positive role of slowing down the damage to chrysanthemum photosynthesis by salt stress.

High salinity would cause lipid peroxidation and bring about the accumulation of MDA, thus MDA content could reflect the degree of plant damage caused by salt stress (Yoshimura et al., 2004). WT chrysanthemum accumulated more MDA than overexpressed lines (**Figure 7A**), demonstrating that *DgWRKY4* might protect chrysanthemum by reducing the MDA accumulation level under salt stress. Excess ROS would cause serious damage to plant protein (Zhang X. et al., 2011), and the antioxidant system of plants plays a dominant role in minimizing cellular damage caused by active oxygen and maintaining a ROS balance (Apel and Hirt, 2004). Analyses showed there was a higher activity of ROS scavengers in overexpressed lines than WT under salinity (**Figures 5B–D**), which were consistent with physiological results, as the expression of antioxidant genes (*DgCuZnSOD*, *DgCAT*, and *DgAPX*) was up regulated under salinity (**Figures 9A–C**). The final result proved that *DgWRKY4* transgenic chrysanthemum exhibited lower ROS accumulation than WT under salt stress (**Figures 7A–D**). Therefore, physiological and molecular double experiments showed that overexpression of *DgWRKY4* was beneficial to ROS-scavenging system to work better, thereby enhancing the salt tolerance of chrysanthemum.

To alleviate the dehydration evoked by high salinity, plants would increase accumulation of metabolites, such as soluble protein, soluble sugar, and proline (Vinocur and Altman, 2005). Among them, proline not only plays important roles in osmotic adjustment, protecting cellular macromolecules and cell membrane structures (Singh et al., 2000) but also scavenging ROS under stresses (Miller et al., 2010). In our study, transgenic chrysanthemum accumulated more proline and soluble sugar than WT under salinity (**Figures 8E,F**). And the expression level of *DgP5CS* was up regulated in overexpressed lines (**Figure 9D**), which was consistent with the increase of proline. All above results suggested that *DgWRKY4* might enhance osmotic regulation ability of transgenic chrysanthemum to resist salt stress.

## Conclusion

In conclusion, our study identified DgWRKY4 as a salt-inducible TF, as well as a positive regulator of salt tolerance in chrysanthemum. The results showed that *DgWRKY4* was up regulated by NaCl, and *DgWRKY4* overexpression improved salt tolerance of transgenic chrysanthemum. The enhanced tolerance of transgenic chrysanthemum was achieved by relatively strong photosynthetic capacity, great activities of antioxidant enzymes, high accumulation of proline and soluble sugar, and improved expression of stress-related genes, suggesting that overexpression of *DgWRKY4* may lead to an effective ROS-scavenging and osmotic adjustment system to maintain cell stability and alleviate the harm of salt stress to plants. Therefore, *DgWRKY4* can serve as an important candidate gene for salt-tolerant plant breeding. Further research will focus on down-stream target genes of *DgWRKY4* to understand its deeper molecular mechanisms in salt stress response.

## Author Contributions

KW, Y-HW, and Q-LL conceived and designed the experiments; KW, Y-HW, Z-YB, Q-LL, and Q-YL performed the experiments; Y-ZP, LZ, B-BJ, and X-QT analyzed the data; KW wrote the paper; and all authors read and approved the manuscript.

## Conflict of Interest Statement

The authors declare that the research was conducted in the absence of any commercial or financial relationships that could be construed as a potential conflict of interest.
